# c-Met信号通道参与HGF诱导不同基因型非小细胞肺癌细胞株对吉非替尼耐药

**DOI:** 10.3779/j.issn.1009-3419.2013.09.05

**Published:** 2013-09-20

**Authors:** 香兰 玄, 昌善 安, 彩存 周

**Affiliations:** 1 133000 延吉，延边大学附属医院呼吸内科 Department of Respiratory Disease, Yanbian University Hospital, Yanji 133000, China; 2 200433 上海，上海同济大学附属肺科医院肿瘤科 Department of Oncology, Tongji University Afliatded Shanghai Pulmonary Hospital, Shanghai 200433, China

**Keywords:** 肝细胞生长因子, 吉非替尼, c-Met, 耐药, 肺肿瘤, HGF, Gefitinib, c-Met, Resistance, Lung neoplasm

## Abstract

**背景与目的:**

肝细胞生长因子（hepatocyte growth factor, HGF）诱导非小细胞肺癌（non-small cell lung cancer, NSCLC）对吉非替尼耐药，可能与其受体c-Met激活有关。本研究旨在探讨c-Met及其下游信号通道是否参与HGF诱导不同基因型NSCLC细胞株对吉非替尼耐药。

**方法:**

选择人NSCLC细胞株表皮生长因子受体(epidermal growth factor receptor, EGFR)突变型PC-9、PC9/R和EGFR野生型H292、A549，用HGF诱导细胞，通过MTT法检测细胞增殖，Annexin V-FITC法检测细胞凋亡，应用免疫印迹技术检测细胞中c-Met及下游通道的变化。

**结果:**

吉非替尼对PC9、H292、A549的生长抑制作用呈浓度依赖性，HGF诱导后吉非替尼抑制细胞的生长曲线明显往右移。在PC9、H292、A549细胞中，吉非替尼和HGF处理组的细胞凋亡率比吉非替尼处理组均减少（*P* < 0.05），在PC9/R细胞中无明显减少（*P* > 0.05）。HGF能激活PC9、H292、PC9/R、A549细胞中c-Met及其下游通道蛋白。在PC9、H292、A549细胞中，吉非替尼和HGF处理组的p-Met、p-Akt、p-Stat3、p-Erk1/2蛋白表达比吉非替尼处理组均增高，在PC9/R细胞中无明显增高。

**结论:**

在体外HGF诱导不同基因型NSCLC细胞株对吉非替尼耐药，c-Met及其下游信号通道参与HGF诱导不同基因型NSCLC细胞株对吉非替尼耐药。

## Abstract (en)

**Background and objective:**

It has been known that hepatocyte growth factor (HGF) induces gefitinib resistance in non-small cell lung cancer (NSCLC) cells. The possible mechanism may be related to the activation of the HGF receptor c-Met. The aim of this study is to investigate the involvement of c-Met and its downstream signaling pathway in the HGF-induced gefitinib resistance of NSCLC cells with different epidermal growth factor receptor (EGFR) gene types.

**Methods:**

NSCLC cell lines with different *EGFR* genes (PC-9, PC9/R, H292, and A549) were selected and induced by HGF. Cell survival was determined by MTT assay and the expression of Met and downstream signaling proteins were examined by Western blot.

**Results:**

Gefitinib inhibited the cell growth of PC9, H292, and A549 cell lines in a dose-dependent manner. The concentration-survival curve notably shifted to the right when induced by HGF. The apoptotic rate was lower when the cells were treated with HGF and gefitinib than when these cells were treated with gefitinib alone (*P* < 0.05), particularly in PC9, H292, and A549 cells, but not in PC9/R. HGF stimulated the phosphorylation of Met and downstream signaling proteins in PC9, H292, PC9/R, and A549 cell lines. p-Met, p-Akt, p-Stat3, and p-Erk1/2 expressions were higher when the cells were treated with HGF and gefitinib than when these cells were treated with gefitinib alone, particularly in PC9, H292, and A549 cells, but not in PC9/R.

**Conclusion:**

c-Met and its downstream signaling pathway possibly participated in the HGF-induced gefitinib resistance in NSCLC cells with different EGFR gene types.

吉非替尼是表皮生长因子受体酪氨酸激酶抑制剂（epidermal growth factor receptor-tyrosine kinase inhibitor, EGFR-TKI），是目前最常用的治疗非小细胞肺癌（non-small cell lung cancer, NSCLC）的分子靶向药物。由于吉非替尼对特殊患者的选择性，仅部分NSCLC患者对吉非替尼有效，存在原发或获得耐药，这种耐药机制不十分明确。新近研究^[1, 2]^显示，肝细胞生长因子（hepatocyte growth factor, HGF）及其受体MET参与NSCLC细胞对吉非替尼耐药。本研究选择NSCLC细胞*EGFR*突变型PC-9、PC9/R和*EGFR*野生型H292、A549，用HGF诱导这4株细胞，应用MTT法检测细胞增殖，Annexin V-FITC法检测细胞凋亡，利用蛋白质免疫印迹技术检测c-Met及下游通道蛋白的表达，验证c-Met及下游信号通道参与HGF诱导不同基因型NSCLC细胞株对吉非替尼耐药。

## 1 材料与方法

### 1.1 材料

人NSCLC细胞株PC9（*EGFR*突变型，敏感株）、H292（*EGFR*野生型，敏感株）、PC9/R（*EGFR*突变型，获得性耐药株）、A549（*EGFR*野生型，原发性耐药株）均由上海市肺科医院中心实验室提供；吉非替尼原料购自济南汇丰达化工有限公司；HGF购自Humanzyme；MTT粉购自AMRESCO；FITC Annexin V Apoptosis Detection Kit 1购自美国BD；兔抗人p-Met（Tyr1349, 145 kDa）、c-Met（190/56 kDa），p-Akt（Ser473, 60 kDa）、Akt（59 kDa），Erk1（44 kDa），p-Stat3（Ser727, 92 kDa）、Stat3（92 kDa），GAPDH（35 kDa）购自EPITOMICS，p-Erk1/2（Tyr202/Y204, 42/44 kDa）购自CST，辣根过氧化物酶标记的羊抗兔二抗购自JECTION；NC膜购自Whatman；ECL化学发光试剂购自Thermo。

### 1.2 细胞培养

分别将PC9、H292、PC9/R、A549细胞常规培养于含10%新生牛血清的DMEM培养液中，置于5%CO_2_、37 ℃恒温细胞培养箱中培养，每3-4天换液传代1次。

### 1.3 MTT法检测细胞增殖

取对数生长期的细胞用胰酶消化，每100 μL含细胞数为5×10^3^个的细胞悬液接种于96孔板。细胞贴壁后，每孔内加入干预药物及细胞因子。72 h后，每孔内加入20 μL MTT（5 mg/mL），放入细胞培养箱中孵育。4 h后，1, 200 rpm、10 min离心，弃上清液，每孔加入200 μL DMSO，摇床上混匀约30 min至结晶完全溶解，用酶标仪测量波长530 nm时OD值。细胞存活率=（实验组平均OD值-空白组平均OD值）/（对照组平均OD值-空白组平均OD值）×100%。实验重复3次，用细胞存活率做出量效曲线。

### 1.4 流式细胞仪检测细胞凋亡

取1×10^5^个对数生长期细胞接种于6孔板，培养24 h。贴壁后弃原培养液，给予相应药物及细胞因子处理。72 h后，胰酶消化并收集全部细胞到5 mL试管，1, 500 rpm、5 min，离心去上清液，生理盐水洗涤1次。加入1×Binding Buffer调整细胞浓度为1×10^6^个/mL，取100 μL（1×10^5^个细胞）到新的5 mL试管。各管内加入5 μL FITC和5 μL PI，室温、避光15 min。上机前加入400 μL 1×Binding Buffer，1 h前进行检测。实验重复3次。

### 1.5 免疫印迹法检测蛋白表达水平

取对数生长的细胞，给予相应处理后立即冰上裂解细胞，4 ℃、12, 000 rpm、30 min、离心收集各组蛋白裂解液。用BCA蛋白定量法定量。取30 μg-40 μg蛋白经8%-10% SDS-PAGE电泳分离后，转印至NC膜上，用5%脱脂奶粉封闭1 h，一抗孵育4 ℃过夜，TBST洗膜10 min、3次后二抗室温摇床孵育1 h，TBST洗膜5 min、5次后ECL化学发光试剂显色、曝光成像。

### 1.6 统计学分析

实验数据用SPSS 17.0统计学软件进行处理。实验数据以均数±标准差表示，组间比较采用*t*检验分析。*P* < 0.05为差异有统计学意义。

## 2 结果

### 2.1 HGF刺激不同时间段c-Met及其下游通道蛋白水平的变化

以40 ng/mL HGF刺激细胞时间段分别为0 min、15 min、30 min、60 min，裂解细胞取蛋白进行蛋白印迹。结果显示，HGF可活化c-Met及其下游通道蛋白磷酸化，随着时间的推移，各细胞中p-Met、p-Akt、p-Stat3、p-Erk1/2蛋白含量出现不同程度的变化。PC9，H292和PC9/R，A549细胞的最佳HGF刺激时间段分别于60 min、30 min、15 min时比其他时间段c-Met及其下游通道磷酸化蛋白表达明显（图 1）。

**1:**
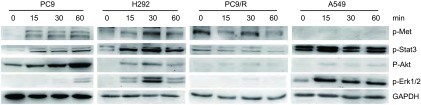
HGF（40 ng/mL）诱导不同时间c-Met及其下游通道磷酸化的表达 The phosphorylation of Met and downstreams signaling pathways induced by HGF (40 ng/mL) in different time

### 2.2 吉非替尼或/和HGF处理的药物浓度-生长曲线

吉非替尼浓度分别取0 μmol/L、0.01 μmol/L、0.04 μmol/L、0.1 μmol/L、0.4 μmol/L、1 μmol/L、4 μmol/L、10 μmol/L、40 μmol/L、100 μmol/L，根据MTT法检测结果，吉非替尼作用于HGF诱导PC9、H292、A549细胞的药物浓度-细胞存活率曲线与非诱导曲线相比明显往右侧移位，PC9/R细胞则未见移位（图 2）。吉非替尼对HGF诱导PC9、H292细胞的半数抑制浓度（IC_50_）至少提高100倍，A549细胞的IC_50_至少提高2倍，PC9/R细胞的IC_50_则无明显差异（表 1）。

**2:**
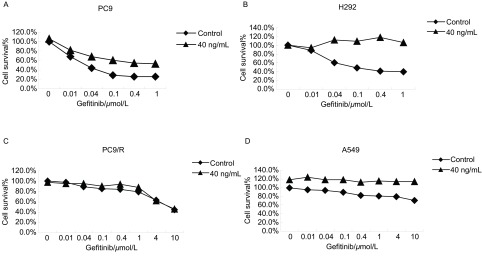
吉非替尼或/和HGF处理的浓度-存活率曲线。A、B、D：HGF诱导时曲线往右移；C：HGF诱导时曲线无右移。 The concentration-survival curve when treated with gefitinib or/and HGF. A, B, D: curve shifts right when induced by HGF; C: curve no shifts right when induced by HGF.

**1:** HGF诱导前后吉非替尼半数抑制浓度（IC_50_） Gefitinib IC_50_ before or after being induced by HGF

	Before being induced (*μ*mol/L)	After being induced (*μ*mol/L)
PC9	0.05±0.02	7.45±1.13^*^
H292	0.11±0.05	> 10
PC9/R	7.19±1.05	8.46±0.64
A549	37.5±1.89	> 100
^*^: compared with that before being induced, *P* < 0.05. HGF: hepatocyte growth factor.		

### 2.3 吉非替尼或/和HGF处理对细胞凋亡的影响

每种细胞分为四个实验组：对照组（C）、HGF组（H）、吉非替尼组（G）、HGF和吉非替尼组（HG）。以40 ng/mL HGF与1 μmol/L吉非替尼单用或联合作用48 h后检测细胞凋亡率。根据表 2显示，PC9、H292、A549细胞的HG组与G组相比，凋亡率明显减少，有统计学意义（*P* < 0.05）。PC9/R细胞的HG组与G组凋亡率无明显差异（*P* > 0.05）。

**2:** HGF诱导耐药对细胞凋亡的影响 The apoptosis in resistance induced by HGF

	C	H	G	HG
PC9	2.4±1.1%	3.3±1.3%	12.1±2.0%	3.2±1.7%^*^
H292	11.2±3.2%	8.7±4.2%	31.8±10.4%	21.5±8.2%^*^
PC9/R	2.6±2.1%	2.1±3.1%	8.4±1.5%	4.7±1.9%
A549	5.6±2.4%	3.0±1.2%	10.8±3.6%	3.1±1.4%^*^
C: control; H: HGF; G: gefitinib; HG: HGF+gefitinb. ^*^: compared with G group, *P* < 0.05.				

### 2.4 吉非替尼或/和HGF处理对细胞c-Met及其下游蛋白表达的影响

首先细胞饥饿过夜，1 μmol/L吉非替尼处理24 h，继续以40 ng/mL HGF刺激PC9细胞1 h、H292和PC9/R细胞30 min、A549细胞15 min后裂解细胞提取蛋白，进行免疫印迹。根据免疫印迹结果，HGF均能刺激PC9、H292、PC9/R、A549细胞中p-Met、p-Akt、p-Stat3、p-Erk1/2的活性。吉非替尼与HGF共同处理时，不同细胞间c-Met及其下游通道蛋白表达有差异。在PC9、H292、A549细胞，吉非替尼均能抑制非HGF诱导细胞内p-Met、p-Akt、p-Stat3、p-Erk1/2的活性，而不能抑制HGF诱导细胞内p-Met、p-Akt、p-Stat3、p-Erk1/2的活性。在PC9/R细胞，吉非替尼均能抑制HGF非诱导及诱导细胞内p-Akt、p-Stat3、p-Erk1/2的活性。在4种细胞中，无论是否HGF诱导，吉非替尼均能抑制ErbB3的活化（图 3）。

**3:**
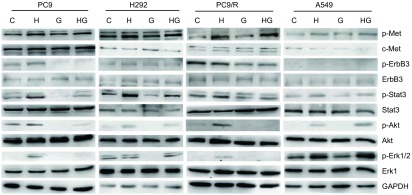
HGF诱导耐药对Met及其下游通道蛋白的影响 The phosphorylation of Met and downstreams signaling pathways in resistance induced by HGF

## 3 讨论

EGFR-TKIs对部分特殊患者有效，非选择的NSCLC患者有效率只有20%^[3]^。*EGFR*基因类型与EGFR-TKIs有效率有很大的相关性，突变型患者中70%-75%有效^[4]^，野生型患者中10%-15%有效^[5]^，存在原发或获得性耐药，提示并非所有突变株对吉非替尼敏感，也并非所有野生株对吉非替尼耐药。本研究选择的4种NSCLC细胞分别具有以下特点：PC9细胞是*EGFR*基因外显子19缺失，故吉非替尼对其IC_50_值为（0.05±0.02）μmol/L；PC9/R细胞是PC9细胞诱导耐药株，同样存在*EGFR*基因外显子19缺失，但吉非替尼对其IC_50_值为（7.19±1.05）μmol/L，比敏感株耐药100倍以上；H292细胞是*EGFR*基因野生型，吉非替尼对其IC_50_值为（0.11±0.05）μmol/L；A549细胞同样是*EGFR*基因野生型，但吉非替尼对其IC_50_值为（37.5±1.89）μmol/L。也就是说PC9和H292细胞是不同*EGFR*基因型的敏感株，PC9/R和A549是不同*EGFR*基因型的耐药株。

*EGFR*二次突变（T790M）和*MET*基因扩增是目前EGFR-TKIs获得性耐药的两大主要分子机制，其他可能的机制有胰岛素样生长因子1受体过表达^[6]^、蛋白酪氨酸磷酸酶基因（*PTEN*）缺失^[7]^、HGF高表达^[8]^等，有待进一步体内外实验研究证实。HGF是成纤维细胞的衍生因子，在NSCLC患者血清中含量明显升高，与肿瘤的侵袭状态密切相关^[9]^。HGF与其特异性受体c-Met结合而发挥作用，异常的HGF/c-Met信号途径与肿瘤细胞生长、运动、粘附、转移、凋亡等因素相关。本研究中，除了PC9/R细胞，吉非替尼对PC9、H292、A549的生长抑制作用呈浓度依赖性，HGF诱导后吉非替尼抑制细胞的生长曲线往右移。HGF诱导后吉非替尼对PC9、H292、A549细胞的IC_50_值明显升高，吉非替尼对HGF诱导的细胞凋亡率比非诱导细胞明显减少。提示HGF明显提高了吉非替尼IC_50_值，使不同基因型肺癌细胞存活率升高。

HGF与c-Met结合后c-Met发生自身磷酸化，激活细胞内各种重要信号通路，最后调控细胞的一系列生命活动。Met下游通道包括MAPK、PI3K/Akt和c-Src/FAK、STAT等通路，其中MAPK通路主要调控细胞分散及增殖，PI3K/Akt通路主要调控细胞活力及存活，c-Src/FAK通路主要调控细胞粘附及转移，STAT通路主要调控细胞形态发生。本文中HGF刺激不同时间段，HGF在短时间（15 min-60 min）内即可活化c-Met、Akt、Stat3、Erk1/2，且其含量高低不一。分别与其他时间段比较，PC9细胞于1 h、H292和PC9/R细胞于30 min、A549细胞于15 min时c-Met及其下游通道蛋白磷酸化含量最高。

本文中，吉非替尼在无HGF刺激下明显抑制PC9、H292、A549细胞细胞内Akt、Stat3、Erk1/2的活化，但在HGF诱导下不能抑制PC9、H292、A549细胞内c-Met、Akt、Stat3、Erk1/2活化，同时检测的非磷酸化的c-Met、Akt、Stat3、Erk1/2总量无明显变化。说明c-Met及其下游通道蛋白磷酸化参与了吉非替尼耐药机制。在PC9/R细胞中，HGF虽能活化c-Met及下游信号蛋白，但与吉非替尼共同处理时不能持续活化c-Met下游通道，这与MTT及凋亡结果相符，其原因有待进一步研究。本文结果提示，HGF诱导的吉非替尼耐药需要上游蛋白的参与，也需要其下游信号蛋白的参与。Bean等^[10]^报道在吉非替尼获得性耐药患者中检测到*MET*基因高扩增，明确*MET*基因扩增激活ErbB3/PI3K/Akt信号途径，绕过吉非替尼治疗的靶点，导致NSCLC对吉非替尼产生耐药。本研究中，虽然HGF诱导c-Met/Akt信号途径活化，但未见ErbB3活化，提示ErbB3可能不参与HGF诱导的不同基因型NSCLC细胞株对吉非替尼耐药。
